# Supplementary material for: Safety and Immunogenicity of a New Rotavirus-Inactivated Vaccine in the Chinese Adolescent Population: A Randomized, Double-Blind, Placebo-Controlled Phase I Clinical Trial
Source: Vaccines (Basel). 2025 Mar 30;13(4):369. doi: 10.3390/vaccines13040369 (PMC12031238; doi:10.3390/vaccines13040369)
Supplement: Supplementary file 1 [file vaccines-13-00369-s001.zip › vaccines-3453731-supplementary.pdf]

Supplementary Data

S1. Development of this inactivated rotavirus vaccine

In this study, a novel inactivated rotavirus vaccine using a human wild-type rotavirus named ZTR-68-A (G1P[8]) isolated from a child's stool with diarrhoea in Zhaotong, Yunnan, China. ZTR-68-A (genotype G1-P[8]-I1-R1-C1-M1-A1-N1-T1-E1-H1) was manufactured by the Institute of Medical Biology, Chinese Academy of Medical Science. The virus was cultivated in Vero cells at an MOI of 0.1 in serum-free MEM medium, clarified by centrifugation, and then concentrated by ultrafiltration. Virus purification was performed using ion exchange chromatography and molecular sieve chromatography. Virus was inactivated with formaldehyde, and produced by adding aluminum hydroxide adjuvant, was assessed the safety and immunogenicity in Chinese adolescent population. Preclinical studies have shown the inactivated rotavirus vaccine can induce serum neutralizing antibodies and provide protection in animal models.

S2. Recruitment Criteria and Exclusion Criteria

2.1. Recruitment Criteria for Vaccine Clinical Trials

2.1.1. Healthy people aged 6–17 years old.

2.1.2. Excluding those with congenital malformations, developmental disorders, genetic defects, severe malnutrition, or other related conditions.

2.1.3. Excluding those with congenital or acquired immunodeficiencies, HIV infection, lymphoma, leukemia, systemic lupus erythematosus (SLE), juvenile rheumatoid arthritis (JRA), or other autoimmune diseases.

2.1.4. Excluding those with a history of cerebral palsy, epilepsy, and psychiatric disorders.

2.1.5. The participation and informed consent of the individual and/or guardian could be obtained and they complied with the clinical trial protocol.

2.1.6. They had not received oral administration of a live attenuated rotavirus vaccine.

2.2. Exclusion Criteria First Dose Screening Criteria:

2.2.1. Axillary temperature >37.0°C prior to vaccination.

2.2.2. Those with a history of or currently with intussusception.

2.2.3. Those with a history of convulsions and seizures, a history of epilepsy or psychosis, and those with these conditions in their family history.

2.2.4. History of vaccination allergy.

2.2.5. Acute exacerbation of various acute illnesses (e.g., fever) or chronic diseases within 3 days prior to receiving the study vaccine.

2.2.6. Received immune-enhancing or immunosuppressive therapy (continuous oral or

intravenous drip for more than 14 days) within 3 months.
2.2.7. Received a live attenuated vaccine within 14 days or other vaccines within 7 days.
2.2.8. History of coagulation abnormalities (e.g., coagulation factor deficiency, coagulopathies).
2.2.9. Primary and secondary immunocompromised states (history of thyroid, pancreas, liver, spleen removal, or need for treatment due to thyroid disease within the past 12 months) and those with clinically significant abnormalities in blood biochemistry-, blood routine-, and urine routine-related indicators prior to vaccination.
2.2.10. Participation in other clinical studies was ongoing or planned in the near future.
2.2.11. Participants who, in the judgment of the investigator, had any other factors that make them unsuitable for participation in clinical trials.
2.3. Exclusion Criteria for Subsequent Doses
2.3.1. Serious adverse reactions after the previous vaccination.
2.3.2. Newly identified or newly occurring adverse events after the first vaccination that did not meet the inclusion criteria or that met the first exclusion criteria for continued participation in the study at the discretion of the investigator.
2.3.3. Received a rotavirus vaccine other than the study vaccine during the study period.
2.3.4. Other reasons for exclusion as determined by the investigator.

S3. Recruitment Procedures

Before the start of the study, the investigator or his/her authorized person will contact the guardians of the volunteers (entrusted persons) by means of distributing recruitment notification letters, face-to-face interviews, telephone calls, etc., and invite volunteers who meet the conditions of recruitment and have good compliance to participate in the study; in the course of the study, real-time adjustments will be made to the recruitment progress according to the progress of the study, so as to ensure that the gender of the participants enrolled in the study is relatively balanced.

S4. Informed Consent Process

Informed consent is the voluntary participation of a volunteer in a clinical trial or the voluntary participation of a ward by the volunteer's guardian (proxy) in a clinical trial. Volunteers and guardians of volunteers must first give informed consent when they arrive at the study site. The researcher will inform the volunteers and/or guardians of the volunteers of the content of the informed consent form for the clinical trial verbally and in writing, and under the condition of voluntary participation, the legal guardian or trustee of the volunteers and the researcher will sign the informed consent form together. The informed consent form shall be signed in duplicate, and the guardian or authorized agent of the volunteer shall keep the duplicate, and the original shall be

kept at the trial site.

S5. Program Violations and Deviations

5.1. The list of protocol violations is as follows (including but not limited to):
5.1.1. Failure to give proper informed consent to participants.
5.1.2. Participants were enrolled in the study without meeting the inclusion criteria or meeting the exclusion criteria.
5.1.3. Participants received the wrong study intervention (e.g., vaccination error).
5.1.4. Failure to report a serious adverse event (SAE) within the required time frame.
5.2. The list of protocol deviations is as follows (included but not limited to):
5.2.1. Failure to administer the experimental vaccine within the window period.
5.2.2. Not collecting blood within the window period.
5.2.3. Failure to meet the required time intervals between vaccinations with other vaccines.

S6. Participants

The study population was 6-17 years of age, with written informed consent approved by the Ethical Review Board, and subject/guardian signing the informed consent form and passing a medical examination and the following inclusion and exclusion criteria before enrollment in the study.

S7. Blinding and randomization

A double-blind experimental design was used. The sponsor provided qualified test vaccine 1, test vaccine 2, test vaccine 3, and a placebo control. The statistician used Stata 13.0 software to randomly blind the test vaccines using block group randomization. The vaccine numbering rule was "Y + 4-digit number." The vaccines were randomly assigned to test vaccine 1, test vaccine 2, test vaccine 3, and placebo control using the consecutive numbering method according to a ratio of 1:1:1:1. The results of randomization of participants into groups and randomization of vaccines were correlated according to four letter codes (A, B, C, and D). The results of the randomization of vaccines into groups were correlated using letter codes (A, B, C, and D).

The statistician imported the results of the randomized grouping of participant and the results of the randomized blinding of vaccines into the central randomization system, which was used to manage the entry and exit of vaccines and the allocation of vaccines during the course of the study. After the successful screening of participants, the researchers at each site participating in the clinical trial logged into the central randomization system to obtain the study number of the participant and the

corresponding vaccine number at the same time. For example, if a participant with a study number of 001 at site 01 is assumed to be randomized to a group with a letter code of A, when assigning vaccines, the central randomization system selects the vaccine with the smallest vaccine number with the letter code A from vaccines assigned to site 01 for vaccination. If the vaccine number has already been used, or the vaccine cannot be used because it has been damaged (e.g., broken packaging), has sediment, has abnormal turbidity, or for other reasons, then the central randomization system will select the vaccine from vaccines assigned to site 01 for use. If the vaccine has already been used, or if the vaccine is damaged (including damaged packaging), has sediment that cannot be shaken in, is abnormally cloudy, or cannot be used, the central randomization system will remove the vaccine with the letter code A and the second smallest vaccine number from the vaccines assigned to site 01, and so on. To maintain blinding in the trial, mapping between the letter code corresponding to the participant number and the letter code corresponding to the vaccine number was known only to the non-blinded personnel and was sealed after the assignment was completed. No other personnel involved in the clinical trial were aware of the mapping relationship between participant numbers and vaccine numbers.

In order to maintain blinding in the trial, the mapping between the letter code corresponding to the subject number and the letter code corresponding to the vaccine number was known only to the non-blinded personnel and was sealed after the assignment was completed. No other personnel involved in the clinical trial were aware of the mapping relationship between subject numbers and vaccine numbers.

Statistical blinders prepare a randomized grouping blinding program to create blind bases. Blinds were categorized into primary and secondary blinds. The primary blind is the group code, each vaccine number, i.e., the study number corresponding to the test or control vaccine, represented by a different letter, and the secondary blind will reveal the final blind, i.e., the name of the vaccine represented by the letter, the test or control vaccine. The randomized group blinding procedure and the blinded bottom are kept in duplicate in separate envelopes, signed and sealed, one for the investigator and one for the sponsor. Blinders shall not participate in the clinical trial and shall not disclose the contents of the blinding to any person participating in the clinical trial.

After completing the 6-month safety observation of the full immunization and obtaining the full immunogenicity result, after consultation and agreement between the sponsor and the researcher, the data manager and the statistician will complete the blind audit of the database, lock the database, and carry out the blinding.

In the event of an emergency situation (e.g. serious adverse event) arising during the fieldwork, the Ethics Committee will be informed and emergency blinding can be carried out if necessary. An online electronic system will be used for emergency

blinding, with one investigator, usually the site manager, authorized at each study site. When a situation requiring emergency blinding occurs at the study site, such as a possible vaccine-related SAE, and the group of vaccines needs to be quickly informed for emergency treatment, the investigator at the site can log in to his/her personal authorization account on the system and initiate an application for emergency blinding, and the system will send the application for emergency blinding to the sponsor, PI, and monitor at the same time, and the authorized personnel of each party will log in to the system for approval and approval. The online system will inform the site manager of the blinding status of the study number. If there is a mass adverse event or any reason to interrupt the trial, the blinding will be released in advance with the joint approval of the sponsor and the investigator.

S8. Assessment of adverse events

8.1 In general reactions, when a subject develops an AE that is consistent with the nature, severity, and frequency of adverse reactions or events mentioned in the current investigator's manual, trial protocol, or product insert, it is reasonable to assume that it is related to the experimental vaccine. Such as fever and localized redness and swelling, which may be accompanied by a combination of symptoms such as general malaise, lethargy, loss of appetite, and malaise.

8.2 Among the abnormal reactions, when a subject develops an AE whose nature, severity, and frequency are not mentioned in the current investigator's manual, trial protocol, or product specification, but which causes a certain degree of damage to the subject's body tissues, organs, and functions, it is judged in principle to be a suspected abnormal reaction to preventive vaccination, which may be related to the test vaccine, and which needs to be investigated and judged by the county or municipal expert group on the investigation and diagnosis of abnormal reactions to preventive vaccination.

S9. Determination of inactivated rotavirus vaccine antigen content

Take the rotavirus antigen detection enzyme plate and add 100 μl of sample dilution to each well. Each ELISA plate can determine three samples to be tested, each take 100 μl of the sample to be tested S1, added to the A1 wells and B1 wells, at this time the total volume of the wells 200 μl, the sample dilution of 1:2. Similarly, take the samples to be tested S2 and S3, respectively, to add to the corresponding C1 - F1 wells. Rotavirus inactivated vaccine finished product take 0.5ml of the sample, add 0.5ml of dissociation solution, at this time, the antigen dilution ratio is 1:2, mixed with room temperature static for 30-35min. disassociation formulations: 20% diethanolamine 1.25ml; 10% Triton-X100 0.2ml; 0.01mol/L PBS 8.55ml. Perform a 2-fold gradient dilution using an 8- or 12-channel pipette from A-F in column 1 to A-F in column 12,

i.e., dilutions of 1:2, 1:4, 1:8, 1:16, 1:32, 1:64, 1:128, 1:256, 1:512, 1:1024, 1:2048, 1:4096. ensure that sample wells are in 100 μl volume solution per well. The 96-well ELISA plate was incubated in an incubator at 37.0℃±1.0℃ for 1 hour. The plate was washed 5 times with ELISA wash solution, 300 μl/well, operated by plate washer. Add HRP-labeled goat anti-rotavirus antibody (Institute of Medical Biology, Chinese Academy of Medical Sciences), corresponding to the ratio of 1 ELISA plate: 5 μl of antibody diluted in 10 ml (dilution ratio of 1:2000) ELISA enzyme-labeled antibody diluent, 100 μl/well, incubate for 1 hour at 37.0℃±1.0℃. The plate was washed 5 times with ELISA wash solution, 300 μl/well, operated by plate washer. Before color development, please make sure that the ELISA plate has been tapped dry solution, please tap dry on filter paper, add TMB, 100 μl/well under the environment of avoiding direct light, the time range of color development is controlled within 2-10 minutes, and then add 100 μl/well ELISA termination solution to terminate the color development. The light absorption values were detected at 450 nm, 650 nm with an enzyme marker. Cutoff value calculation: Cutoff threshold=mean OD value of negative control×2.1. If the mean negative control OD value is <0.050, the Cutoff threshold is calculated as 0.050. When the OD of the sample to be tested is higher than the Cutoff value, the sample to be tested is considered positive, and the amount of antigen (EU/ml) is read as the maximum dilution of the positive result x 10. The geometric mean of the two results is taken as the final result.

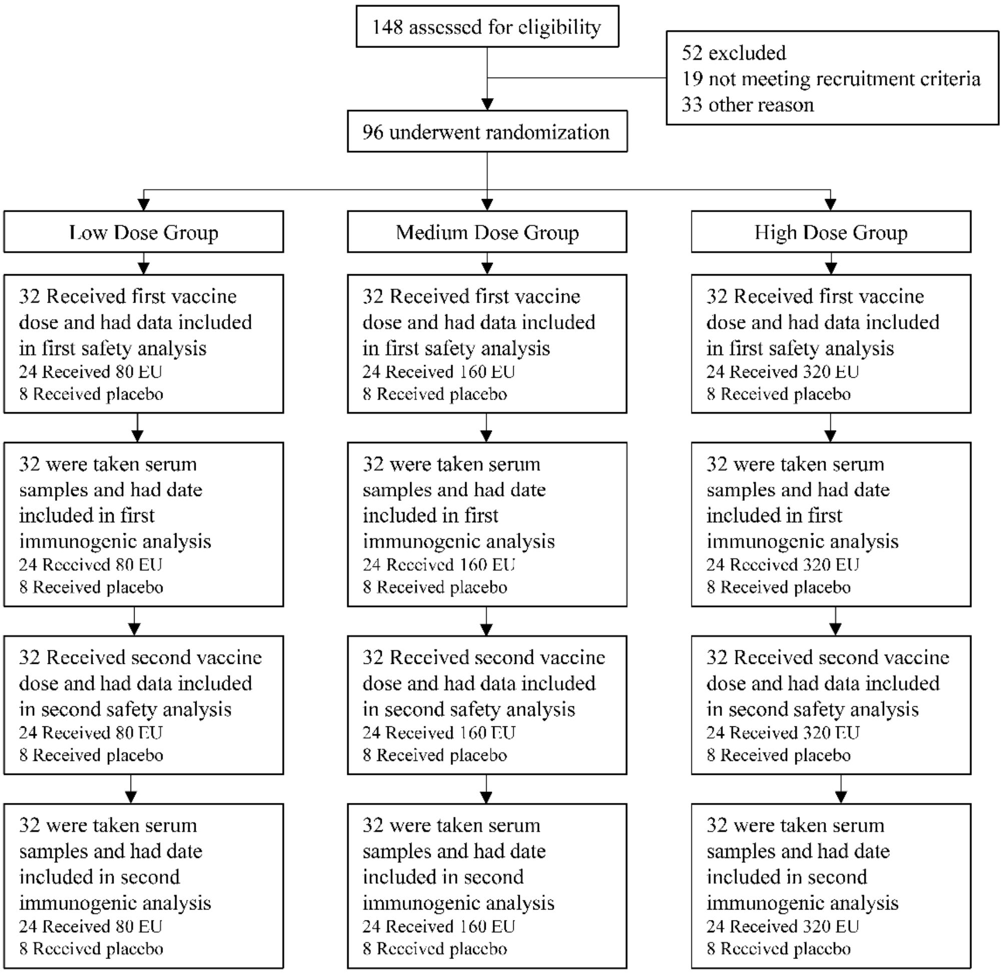

Figure S1. Flow chart of participants through the Trail

148 assessed for eligibility

↓

96 underwent randomization

↓

3 Dose Groups

↓

Days	Vaccine Injection	Security Observation	AE Record	Immunogenicity Assay
0	Yes	Blood biochemistry, blood routine, urine routine	1、Adverse events from 0 to 7 days after vaccination and instructed participants to record daily temperature measurements and adverse events on a diary card for initial safety assessment on day 8 after the first dose of vaccine. 2、All participants continued to be observed for adverse events from 8 to 30 days and records were kept. 3、Participants were required to continue follow-up up to 6 months after full vaccination, with only serious adverse events (SAEs) recorded.	Neutralizing antibody, IgG, IgA
4 days after first injection		Blood biochemistry, blood routine, urine routine		Neutralizing antibody, IgG, IgA
14 days after first injection				Neutralizing antibody, IgG, IgA
28	Yes	Blood biochemistry, blood routine, urine routine		Neutralizing antibody, IgG, IgA
4 days after final injection		Blood biochemistry, blood routine, urine routine		Neutralizing antibody, IgG, IgA
14 days after final injection				Neutralizing antibody, IgG, IgA
28 days after final injection				Neutralizing antibody, IgG, IgA
30 days after final injection				
90 days after final injection				Neutralizing antibody, IgG, IgA
180 days after final injection				Neutralizing antibody, IgG, IgA

Figure S2. Vaccination scheme

Table S1. Incidence of adverse events/reactions sorted by type in the 6-17 age group 0-30 days after full vaccination with the test vaccine and placebo

Type	Groups	N	Correlation					Irrelevance					Total				
			Number of occurrences	Number of patients	Rate (95% CI)	X^2	*P*	Number of occurrences	Number of patients	Rate (95% Ci)	X^2	*P*	Number of occurrences	Number of patients	Rate (95% CI)	X^2	*P*
Total adverse events	Placebo	24	3	2	8.33(1.03-27.00)			17	6	25.00(9.77-46.71)			20	6	25.00(9.77-46.71)		
	Low Dose	24	12	9	37.50(18.80-59.41)	5.779	0.016[a]	13	10	41.67(22.11-63.36)	1.500	0.221[a]	25	13	54.17(32.82-74.45)	4.269	0.039a
	Medium Dose	24	5	3	12.50(2.66-32.36)	<0.001	>0.999[b]	7	5	20.83(7.13-42.15)	0.118	0.731[b]	12	7	29.17(12.62-51.09)	0.105	0.745b
	High Dose	24	2	1	4.17(0.11-21.12)	<0.001	>0.999[c]	7	5	20.83(7.13-42.15)	0.118	0.731[c]	9	5	20.83(7.13-42.15)	0.118	0.731c
Local adverse event	Placebo	24	1	1	4.17(0.11-21.12)			0	0	0.00(0.00-14.25)			1	1	4.17(0.11-21.12)		
	Low Dose	24	6	6	25.00(9.77-46.71)	2.676	0.102[a]	0	0	0.00(0.00-14.25)			6	6	25.00(9.77-46.71)	2.676	0.102[a]
	Medium Dose	24	2	1	4.17(0.11-21.12)	<0.001	>0.999[b]	0	0	0.00(0.00-14.25)			2	1	4.17(0.11-21.12)	<0.001	>0.999[b]
	High Dose	24	2	1	4.17(0.11-21.12)	<0.001	>0.999[c]	0	0	0.00(0.00-14.25)			2	1	4.17(0.11-21.12)	<0.001	>0.999[c]
Systemic adverse event	Placebo	24	2	2	8.33(1.03-27.00)			17	6	25.00(9.77-46.71)			19	6	25.00(9.77-46.71)		
	Low Dose	24	6	4	16.67(4.74-37.38)	0.190	0.663[a]	13	10	41.67(22.11-63.36)	1.500	0.221[a]	19	12	50.00(29.12-70.88)	3.200	0.074[a]
	Medium Dose	24	3	3	12.50(2.66-32.36)	<0.001	>0.999[b]	7	5	20.83(7.13-42.15)	0.118	0.731[b]	10	7	29.17(12.62-51.09)	0.105	0.745[b]
	High Dose	24	0	0	0.00(0.00-14.25)	0.522	0.470[c]	7	5	20.83(7.13-42.15)	0.118	0.731[c]	7	5	20.83(7.13-42.15)	0.118	0.731[c]
Level 3 and above adverse events	Placebo	24	0	0	0.00(0.00-14.25)			1	1	4.17(0.11-21.12)			1	1	4.17(0.11-21.12)		
	Low Dose	24	0	0	0.00(0.00-14.25)			0	0	0.00(0.00-14.25)		>0.999[a]	0	0	0.00(0.00-14.25)		>0.999[a]
	Medium Dose	24	0	0	0.00(0.00-14.25)			0	0	0.00(0.00-14.25)		>0.999[b]	0	0	0.00(0.00-14.25)		>0.999[b]
	High Dose	24	0	0	0.00(0.00-14.25)			1	1	4.17(0.11-21.12)	<0.001	>0.999[c]	1	1	4.17(0.11-21.12)	<0.001	>0.999[c]

CI: Confidence Interval.

[a] indicates the low dose group compared with the placebo group.

[b] indicates the medium dose group compared with the placebo group.

[c] indicates the high dose group compared with the placebo group.

Table S2. Incidence of adverse events/reactions by type in the 6-17 age group within 30min after full vaccination with the test vaccine and placebo

Type	Groups	N	Correlation					Irrelevance					Total				
			**Number of occurrences**	**Number of patients**	**Rate (95% CI)**	X^2	***P***	**Number of occurrences**	**Number of patients**	**Rate (95% CI)**	X^2	***P***	**Number of occurrences**	**Number of patients**	**Rate (95% CI)**	X^2	***P***
Total adverse events	Placebo	24	0	0	0.00(0.00-14.25)			0	0	0.00(0.00-14.25)			0	0	0.00(0.00-14.25)		
	Low Dose	24	3	3	12.50(2.66-32.36)	1.422	0.233[a]	0	0	0.00(0.00-14.25)			3	3	12.50(2.66-32.36)	1.422	0.233[a]
	Medium Dose	24	0	0	0.00(0.00-14.25)			0	0	0.00(0.00-14.25)			0	0	0.00(0.00-14.25)		
	High Dose	24	0	0	0.00(0.00-14.25)			0	0	0.00(0.00-14.25)			0	0	0.00(0.00-14.25)		
Local adverse event	Placebo	24	0	0	0.00(0.00-14.25)			0	0	0.00(0.00-4.25)			0	0	0.00(0.00-14.25)		
	Low Dose	24	3	3	12.50(2.66-32.36)	1.422	0.233[a]	0	0	0.00(0.00-14.25)			3	3	12.50(2.66-32.36)	1.422	0.233[a]
	Medium Dose	24	0	0	0.00(0.00-14.25)			0	0	0.00(0.00-14.25)			0	0	0.00(0.00-14.25)		
	High Dose	24	0	0	0.00(0.00-14.25)			0	0	0.00(0.00-14.25)			0	0	0.00(0.00-14.25)		
Systemic adverse event	Placebo	24	0	0	0.00(0.00-14.25)			0	0	0.00 (0.00-14.25)			0	0	0.00 (0.00-14.25)		
	Low Dose	24	0	0	0.00(0.00-14.25)			0	0	0.00 (0.00-14.25)			0	0	0.00 (0.00-14.25)		
	Medium Dose	24	0	0	0.00(0.00-14.25)			0	0	0.00 (0.00-14.25)			0	0	0.00(0.00-14.25)		
	High Dose	24	0	0	0.00(0.00-14.25)			0	0	0.00 (0.00-14.25)			0	0	0.00(0.00-14.25)		

CI: Confidence Interval.

[a] indicates the low dose group compared with the placebo group.

[b] indicates the medium dose group compared with the placebo group.

[c] indicates the high dose group compared with the placebo group.

Table S3. Incidence of symptomatic adverse events/reactions within 0-30 days after full vaccination with the test vaccine and placebo in the 6-17 age group

SOC	PT	Groups	N	Correlation					Irrelevance					Total				
				Number of occurrences	Number of patients	Rate (95% CI)	X^2	*P*	Number of occurrences	Number of patients	Rate (95% CI)	X^2	*P*	Number of occurrences	Number of patients	Rate (95% CI)	X^2	*P*
Infections and infestations	Summary	Placebo	24	0	0	0.00 (0.00-14.25)			3	3	12.50 (2.66-32.36)			3	3	12.50 (2.66-32.36)		
		Low Dose	24	0	0	0.00 (0.00-14.25)			2	2	8.33 (1.03-27.00)	<0.001	>0.999[a]	2	2	8.33 (1.03-27.00)	<0.001	>0.999[a]
		Medium Dose	24	0	0	0.00 (0.00-14.25)			1	1	4.17 (0.11-21.12)	0.273	0.602[b]	1	1	4.17 (0.11-21.12)	0.273	0.602[b]
		High Dose	24	0	0	0.00 (0.00-14.25)			0	0	0.00 (0.00-14.25)	1.422	0.233[c]	0	0	0.00 (0.00-14.25)	1.422	0.233[c]
	Pneumonia	Placebo	24	0	0	0.00 (0.00-14.25)			0	0	0.00 (0.00-14.25)			0	0	0.00 (0.00-14.25)		
		Low Dose	24	0	0	0.00 (0.00-14.25)			1	1	4.17 (0.11-21.12)		>0.999[a]	1	1	4.17 (0.11-21.12)		>0.999[a]
		Medium Dose	24	0	0	0.00 (0.00-14.25)			0	0	0.00 (0.00-14.25)			0	0	0.00 (0.00-14.25)		
		High Dose	24	0	0	0.00 (0.00-14.25)			0	0	0.00 (0.00-14.25)			0	0	0.00 (0.00-14.25)		
	Upper respiratory tract infections	Placebo	24	0	0	0.00 (0.00-14.25)			3	3	12.50 (2.66-32.36)			3	3	12.50(2.66-32.36)		
		Low Dose	24	0	0	0.00 (0.00-14.25)			1	1	4.17 (0.11-21.12)	0.273	0.602[a]	1	1	4.17 (0.11-21.12)	0.273	0.602[a]
		Medium Dose	24	0	0	0.00 (0.00-14.25)			0	0	0.00 (0.00-14.25)	1.422	0.233[b]	0	0	0.00 (0.00-14.25)	1.422	0.233[b]
		High Dose	24	0	0	0.00 (0.00-14.25)			0	0	0.00 (0.00-14.25)	1.422	0.233[c]	0	0	0.00 (0.00-14.25)	1.422	0.233[c]
	Chickenpox	Placebo	24	0	0	0.00 (0.00-14.25)			0	0	0.00 (0.00-14.25)			0	0	0.00 (0.00-14.25)		

SOC	PT	Groups	N	Correlation					Irrelevance					Total				
				Number of occurrences	Number of patients	Rate (95% CI)	X^2	*P*	Number of occurrences	Number of patients	Rate (95% CI)	X^2	*P*	Number of occurrences	Number of patients	Rate (95% CI)	X^2	*P*
		Low Dose	24	0	0	0.00 (0.00-14.25)			0	0	0.00 (0.00-14.25)			0	0	0.00 (0.00-14.25)		
		Medium Dose	24	0	0	0.00 (0.00-14.25)			1	1	4.17 (0.11-21.12)		>0.999[b]	1	1	4.17 (0.11-21.12)		>0.999[b]
		High Dose	24	0	0	0.00 (0.00-14.25)			0	0	0.00 (0.00-14.25)			0	0	0.00 (0.00-14.25)		
Investigations	Summary	Placebo	24	0	0	0.00 (0.00-14.25)			7	4	16.67 (4.74-37.38)			7	4	16.67 (4.74-37.38)		
		Low Dose	24	0	0	0.00 (0.00-14.25)			4	4	16.67 (4.74-37.38)	<0.001	>0.999[a]	4	4	16.67 (4.74-37.38)	<0.001	>0.999[a]
		Medium Dose	24	0	0	0.00 (0.00-14.25)			0	0	0.00 (0.00-14.25)	2.455	0.117[b]	0	0	0.00 (0.00-14.25)	2.455	0.117[b]
		High Dose	24	0	0	0.00 (0.00-14.25)			6	5	20.83 (7.13-42.15)	<0.001	>0.999[c]	6	5	20.83 (7.13-42.15)	<0.001	>0.999[c]
	White blood cell count abnormal	Placebo	24	0	0	0.00 (0.00-14.25)			3	3	12.50 (2.66-32.36)			3	3	12.50 (2.66-32.36)		
		Low Dose	24	0	0	0.00 (0.00-14.25)			3	3	12.50 (2.66-32.36)	<0.001	>0.999[a]	3	3	12.50 (2.66-32.36)	<0.001	>0.999[a]
		Medium Dose	24	0	0	0.00 (0.00-14.25)			0	0	0.00 (0.00-14.25)	1.422	0.233[b]	0	0	0.00 (0.00-14.25)	1.422	0.233[b]
		High Dose	24	0	0	0.00 (0.00-14.25)			1	1	4.17 (0.11-21.12)	0.273	0.602[c]	1	1	4.17 (0.11-21.12)	0.273	0.602[c]
	Alanine aminotransferase abnormal	Placebo	24	0	0	0.00 (0.00-14.25)			1	1	4.17 (0.11-21.12)			1	1	4.17(0.11-21.12)		
		Low Dose	24	0	0	0.00 (0.00-14.25)			0	0	0.00 (0.00-14.25)		>0.999[a]	0	0	0.00 (0.00-14.25)		>0.999[a]
		Medium Dose	24	0	0	0.00 (0.00-14.25)			0	0	0.00 (0.00-14.25)		>0.999[b]	0	0	0.00 (0.00-14.25)		>0.999[b]
		High Dose	24	0	0	0.00 (0.00-14.25)			1	1	4.17 (0.11-21.12)	<0.001	>0.999[c]	1	1	4.17 (0.11-21.12)	<0.001	>0.999[c]
	Lymphocyte count abnormal	Placebo	24	0	0	0.00 (0.00-14.25)			0	0	0.00 (0.00-14.25)			0	0	0.00 (0.00-14.25)		

SOC	PT	Groups	N	Correlation					Irrelevance					Total				
				Number of occurrences	Number of patients	Rate (95% CI)	X^2	*P*	Number of occurrences	Number of patients	Rate (95% CI)	X^2	*P*	Number of occurrences	Number of patients	Rate (95% CI)	X^2	*P*
		Low Dose	24	0	0	0.00 (0.00-14.25)			0	0	0.00 (0.00-14.25)			0	0	0.00 (0.00-14.25)		
		Medium Dose	24	0	0	0.00 (0.00-14.25)			0	0	0.00 (0.00-14.25)			0	0	0.00 (0.00-14.25)		
		High Dose	24	0	0	0.00 (0.00-14.25)			1	1	4.17 (0.11-21.12)		>0.999[c]	1	1	4.17 (0.11-21.12)		>0.999[c]
	Protein urine present	Placebo	24	0	0	0.00 (0.00-14.25)			1	1	4.17 (0.11-21.12)			1	1	4.17(0.11-21.12)		
		Low Dose	24	0	0	0.00 (0.00-14.25)			0	0	0.00 (0.00-14.25)		>0.999[a]	0	0	0.00(0.00-14.25)		>0.999[a]
		Medium Dose	24	0	0	0.00 (0.00-14.25)			0	0	0.00 (0.00-14.25)		>0.999[b]	0	0	0.00(0.00-14.25)		>0.999[b]
		High Dose	24	0	0	0.00 (0.00-14.25)			2	2	8.33 (1.03-27.00)	<0.001	>0.999[c]	2	2	8.33(1.03-27.00)	<0.001	>0.999[c]
	Red blood cells urine positive	Placebo	24	0	0	0.00 (0.00-14.25)			0	0	0.00 (0.00-14.25)			0	0	0.00 (0.00-14.25)		
		Low Dose	24	0	0	0.00 (0.00-14.25)			1	1	4.17 (0.11-21.12)		>0.999[a]	1	1	4.17 (0.11-21.12)		>0.999[a]
		Medium Dose	24	0	0	0.00 (0.00-14.25)			0	0	0.00 (0.00-14.25)			0	0	0.00 (0.00-14.25)		
		High Dose	24	0	0	0.00 (0.00-14.25)			1	1	4.17 (0.11-21.12)		>0.999[c]	1	1	4.17 (0.11-21.12)		>0.999[c]
	Glucose urine present	Placebo	24	0	0	0.00 (0.00-14.25)			1	1	4.17 (0.11-21.12)			1	1	4.17 (0.11-21.12)		
		Low Dose	24	0	0	0.00 (0.00-14.25)			0	0	0.00 (0.00-14.25)		>0.999[a]	0	0	0.00 (0.00-14.25)		>0.999[a]
		Medium Dose	24	0	0	0.00 (0.00-14.25)			0	0	0.00 (0.00-14.25)		>0.999[b]	0	0	0.00 (0.00-14.25)		>0.999[b]
		High Dose	24	0	0	0.00 (0.00-14.25)			0	0	0.00 (0.00-14.25)		>0.999[c]	0	0	0.00 (0.00-14.25)		>0.999[c]
	Aspartate aminotransferase	Placebo	24	0	0	0.00 (0.00-14.25)			1	1	4.17 (0.11-21.12)			1	1	4.17 (0.11-21.12)		

SOC	PT	Groups	N	Correlation					Irrelevance					Total				
				Number of occurrences	Number of patients	Rate (95% CI)	X^2	P	Number of occurrences	Number of patients	Rate (95% CI)	X^2	P	Number of occurrences	Number of patients	Rate (95% CI)	X^2	P
		Low Dose	24	0	0	0.00 (0.00-14.25)			0	0	0.00 (0.00-14.25)		>0.999[a]	0	0	0.00 (0.00-14.25)		>0.999[a]
		Medium Dose	24	0	0	0.00 (0.00-14.25)			0	0	0.00 (0.00-14.25)		>0.999[b]	0	0	0.00 (0.00-14.25)		>0.999[b]
		High Dose	24	0	0	0.00 (0.00-14.25)			0	0	0.00 (0.00-14.25)		>0.999[c]	0	0	0.00 (0.00-14.25)		>0.999[c]
Respiratory, thoracic and mediastinal disorders	Summary	Placebo	24	1	1	4.17 (0.11-21.12)			5	2	8.33 (1.03-27.00)			6	3	12.50 (2.66-32.36)		
		Low Dose	24	1	1	4.17 (0.11-21.12)	<0.001	>0.999[a]	4	3	12.50 (2.66-32.36)	<0.001	>0.999[a]	5	4	16.67 (4.74-37.38)	<0.001	>0.999[a]
		Medium Dose	24	0	0	0.00 (0.00-14.25)		>0.999[b]	3	2	8.33 (1.03-27.00)	<0.001	>0.999[b]	3	2	8.33 (1.03-27.00)	<0.001	>0.999[b]
		High Dose	24	0	0	0.00 (0.00-14.25)		>0.999[c]	1	1	4.17 (0.11-21.12)	<0.001	>0.999[c]	1	1	4.17 (0.11-21.12)	0.273	0.602[c]
	Nasal obstruction	Placebo	24	0	0	0.00 (0.00-14.25)			0	0	0.00 (0.00-14.25)			0	0	0.00 (0.00-14.25)		
		Low Dose	24	0	0	0.00 (0.00-14.25)			2	2	8.33 (1.03-27.00)	0.522	0.470[a]	2	2	8.33 (1.03-27.00)	0.522	0.470[a]
		Medium Dose	24	0	0	0.00 (0.00-14.25)			0	0	0.00 (0.00-14.25)			0	0	0.00 (0.00-14.25)		
		High Dose	24	0	0	0.00 (0.00-14.25)			0	0	0.00 (0.00-14.25)			0	0	0.00 (0.00-14.25)		
	Cough	Placebo	24	1	1	4.17 (0.11-21.12)			2	1	4.17 (0.11-21.12)			3	2	8.33 (1.03-27.00)		
		Low Dose	24	1	1	4.17 (0.11-21.12)	<0.001	>0.999[a]	1	1	4.17 (0.11-21.12)	<0.001	>0.999[a]	2	2	8.33 (1.03-27.00)	<0.001	>0.999[a]
		Medium Dose	24	0	0	0.00 (0.00-14.25)		>0.999[b]	2	2	8.33 (1.03-27.00)	<0.001	>0.999[b]	2	2	8.33 (1.03-27.00)	<0.001	>0.999[b]
		High Dose	24	0	0	0.00 (0.00-14.25)		>0.999[c]	0	0	0.00 (0.00-14.25)		>0.999[c]	0	0	0.00 (0.00-14.25)	0.522	0.470[c]
	Productive cough	Placebo	24	0	0	0.00 (0.00-14.25)			0	0	0.00 (0.00-14.25)			0	0	0.00 (0.00-14.25)		

SOC	PT	Groups	N	Correlation					Irrelevance					Total				
				Number of occurrences	Number of patients	Rate (95% CI)	X^2	***P***	Number of occurrences	Number of patients	Rate (95% CI)	X^2	***P***	Number of occurrences	Number of patients	Rate (95% CI)	X^2	***P***
		Low Dose	24	0	0	0.00 (0.00-14.25)			0	0	0.00 (0.00-14.25)			0	0	0.00 (0.00-14.25)		
		Medium Dose	24	0	0	0.00 (0.00-14.25)			1	1	4.17 (0.11-21.12)		>0.999[b]	1	1	4.17 (0.11-21.12)		>0.999[b]
		High Dose	24	0	0	0.00 (0.00-14.25)			0	0	0.00 (0.00-14.25)			0	0	0.00 (0.00-14.25)		
	Oropharyngeal pain	Placebo	24	0	0	0.00 (0.00-14.25)			2	2	8.33 (1.03-27.00)			2	2	8.33 (1.03-27.00)		
		Low Dose	24	0	0	0.00 (0.00-14.25)			0	0	0.00 (0.00-14.25)	0.522	0.470[a]	0	0	0.00 (0.00-14.25)	0.522	0.470[a]
		Medium Dose	24	0	0	0.00 (0.00-14.25)			0	0	0.00 (0.00-14.25)	0.522	0.470[b]	0	0	0.00 (0.00-14.25)	0.522	0.470[b]
		High Dose	24	0	0	0.00 (0.00-14.25)			0	0	0.00 (0.00-14.25)	0.522	0.470[c]	0	0	0.00 (0.00-14.25)	0.522	0.470[c]
	Rhinorrhoea	Placebo	24	0	0	0.00 (0.00-14.25)			0	0	0.00 (0.00-14.25)			0	0	0.00 (0.00-14.25)		
		Low Dose	24	0	0	0.00 (0.00-14.25)			1	1	4.17 (0.11-21.12)		>0.999[a]	1	1	4.17 (0.11-21.12)		>0.999[a]
		Medium Dose	24	0	0	0.00 (0.00-14.25)			0	0	0.00 (0.00-14.25)			0	0	0.00 (0.00-14.25)		
		High Dose	24	0	0	0.00 (0.00-14.25)			1	1	4.17 (0.11-21.12)		>0.999[c]	1	1	4.17 (0.11-21.12)		>0.999[c]
	Sputum increased	Placebo	24	0	0	0.00 (0.00-14.25)			1	1	4.17 (0.11-21.12)			1	1	4.17 (0.11-21.12)		
		Low Dose	24	0	0	0.00 (0.00-14.25)			0	0	0.00 (0.00-14.25)		>0.999[a]	0	0	0.00 (0.00-14.25)		>0.999[a]
		Medium Dose	24	0	0	0.00 (0.00-14.25)			0	0	0.00 (0.00-14.25)		>0.999[b]	0	0	0.00 (0.00-14.25)		>0.999[b]
		High Dose	24	0	0	0.00 (0.00-14.25)			0	0	0.00 (0.00-14.25)		>0.999[c]	0	0	0.00 (0.00-14.25)		>0.999[c]
Skin and subcutaneous tissue disorders	Summary	Placebo	24	0	0	0.00 (0.00-14.25)			1	1	4.17 (0.11-21.12)			1	1	4.17 (0.11-21.12)		

SOC	PT	Groups	N	Correlation					Irrelevance					Total				
				Number of occurrences	Number of patients	Rate (95% CI)	X^2	*P*	Number of occurrences	Number of patients	Rate (95% CI)	X^2	*P*	Number of occurrences	Number of patients	Rate (95% CI)	X^2	*P*
		Low Dose	24	0	0	0.00 (0.00-14.25)			1	1	4.17 (0.11-21.12)	<0.001	>0.999[a]	1	1	4.17 (0.11-21.12)	<0.001	>0.999[a]
		Medium Dose	24	0	0	0.00 (0.00-14.25)			0	0	0.00 (0.00-14.25)		>0.999[b]	0	0	0.00 (0.00-14.25)		>0.999[b]
		High Dose	24	0	0	0.00 (0.00-14.25)			0	0	0.00 (0.00-14.25)		>0.999[c]	0	0	0.00 (0.00-14.25)		>0.999[c]
	Rash	Placebo	24	0	0	0.00 (0.00-14.25)			1	1	4.17 (0.11-21.12)			1	1	4.17(0.11-21.12)		
		Low Dose	24	0	0	0.00 (0.00-14.25)			0	0	0.00 (0.00-14.25)		>0.999[a]	0	0	0.00 (0.00-14.25)		>0.999[a]
		Medium Dose	24	0	0	0.00 (0.00-14.25)			0	0	0.00 (0.00-14.25)		>0.999[b]	0	0	0.00 (0.00-14.25)		>0.999[b]
		High Dose	24	0	0	0.00 (0.00-14.25)			0	0	0.00 (0.00-14.25)		>0.999[c]	0	0	0.00 (0.00-14.25)		>0.999[c]
	Eczema	Placebo	24	0	0	0.00 (0.00-14.25)			0	0	0.00 (0.00-14.25)			0	0	0.00 (0.00-14.25)		
		Low Dose	24	0	0	0.00 (0.00-14.25)			1	1	4.17 (0.11-21.12)		>0.999[a]	1	1	4.17 (0.11-21.12)		>0.999[a]
		Medium Dose	24	0	0	0.00 (0.00-14.25)			0	0	0.00 (0.00-14.25)			0	0	0.00 (0.00-14.25)		
		High Dose	24	0	0	0.00 (0.00-14.25)			0	0	0.00 (0.00-14.25)			0	0	0.00 (0.00-14.25)		
General disorders and administration site conditions	Summary	Placebo	24	1	1	4.17 (0.11-21.12)			1	1	4.17 (0.11-21.12)			2	2	8.33 (1.03-27.00)		
		Low Dose	24	9	8	33.33 (15.63-55.32)	4.923	0.027[a]	0	0	0.00 (0.00-14.25)		>0.999[a]	9	8	33.33 (15.63-55.32)	4.547	0.033[a]
		Medium Dose	24	4	3	12.50 (2.66-32.36)	0.273	0.602[b]	1	1	4.17 (0.11-21.12)	<0.001	>0.999[b]	5	3	12.50 (2.66-32.36)	<0.001	>0.999[b]
		High Dose	24	2	1	4.17 (0.11-21.12)	<0.001	>0.999[c]	0	0	0.00 (0.00-14.25)		>0.999[c]	2	1	4.17 (0.11-21.12)	<0.001	>0.999[c]
	Pyrexia	Placebo	24	0	0	0.00 (0.00-14.25)			1	1	4.17 (0.11-21.12)			1	1	4.17 (0.11-21.12)		

SOC	PT	Groups	N	Correlation					Irrelevance					Total				
				Number of occurrences	Number of patients	Rate (95% CI)	X^2	*P*	Number of occurrences	Number of patients	Rate (95% CI)	X^2	*P*	Number of occurrences	Number of patients	Rate (95% CI)	X^2	*P*
		Low Dose	24	3	2	8.33 (1.03-27.00)	0.522	0.470[a]	0	0	0.00 (0.00-14.25)		>0.999[a]	3	2	8.33 (1.03-27.00)	<0.001	>0.999[a]
		Medium Dose	24	1	1	4.17 (0.11-21.12)		>0.999[b]	1	1	4.17 (0.11-21.12)	<0.001	>0.999[b]	2	2	8.33 (1.03-27.00)	<0.001	>0.999[b]
		High Dose	24	0	0	0.00 (0.00-14.25)			0	0	0.00 (0.00-14.25)		>0.999[c]	0	0	0.00 (0.00-14.25)		>0.999[c]
	Asthenia	Placebo	24	0	0	0.00 (0.00-14.25)			0	0	0.00 (0.00-14.25)			0	0	0.00 (0.00-14.25)		
		Low Dose	24	0	0	0.00 (0.00-14.25)			0	0	0.00 (0.00-14.25)			0	0	0.00 (0.00-14.25)		
		Medium Dose	24	1	1	4.17 (0.11-21.12)		>0.999[b]	0	0	0.00 (0.00-14.25)			1	1	4.17 (0.11-21.12)		>0.999[b]
		High Dose	24	0	0	0.00 (0.00-14.25)			0	0	0.00 (0.00-14.25)			0	0	0.00 (0.00-14.25)		
	Vaccination site erythema	Placebo	24	0	0	0.00 (0.00-14.25)			0	0	0.00 (0.00-14.25)			0	0	0.00 (0.00-14.25)		
		Low Dose	24	0	0	0.00 (0.00-14.25)			0	0	0.00 (0.00-14.25)			0	0	0.00 (0.00-14.25)		
		Medium Dose	24	1	1	4.17 (0.11-21.12)		>0.999[b]	0	0	0.00 (0.00-14.25)			1	1	4.17 (0.11-21.12)		>0.999[b]
		High Dose	24	0	0	0.00 (0.00-14.25)			0	0	0.00 (0.00-14.25)			0	0	0.00 (0.00-14.25)		
	Site of vaccination painful	Placebo	24	1	1	4.17 (0.11-21.12)			0	0	0.00 (0.00-14.25)			1	1	4.17 (0.11-21.12)		
		Low Dose	24	6	6	25.00 (9.77-46.71)	2.676	0.102[a]	0	0	0.00 (0.00-14.25)			6	6	25.00 (9.77-46.71)	2.676	0.102[a]
		Medium Dose	24	0	0	0.00 (0.00-14.25)		>0.999[b]	0	0	0.00 (0.00-14.25)			0	0	0.00 (0.00-14.25)		>0.999[b]
		High Dose	24	2	1	4.17 (0.11-21.12)	<0.001	>0.999[c]	0	0	0.00 (0.00-14.25)			2	1	4.17 (0.11-21.12)	<0.001	>0.999[c]
	Vaccination site swelling	Placebo	24	0	0	0.00 (0.00-14.25)			0	0	0.00 (0.00-14.25)			0	0	0.00 (0.00-14.25)		

SOC	PT	Groups	N	Correlation					Irrelevance					Total				
				Number of occurrences	Number of patients	Rate (95% CI)	X^2	*P*	Number of occurrences	Number of patients	Rate (95% CI)	X^2	*P*	Number of occurrences	Number of patients	Rate (95% CI)	X^2	*P*
		Low Dose	24	0	0	0.00 (0.00-14.25)			0	0	0.00 (0.00-14.25)			0	0	0.00 (0.00-14.25)		
		Medium Dose	24	1	1	4.17 (0.11-21.12)		>0.999[b]	0	0	0.00 (0.00-14.25)			1	1	4.17 (0.11-21.12)		>0.999[b]
		High Dose	24	0	0	0.00 (0.00-14.25)			0	0	0.00 (0.00-14.25)			0	0	0.00 (0.00-14.25)		
Gastrointestinal disorders	Summary	Placebo	24	1	1	4.17 (0.11-21.12)			0	0	0.00 (0.00-14.25)			1	1	4.17 (0.11-21.12)		
		Low Dose	24	2	1	4.17 (0.11-21.12)	<0.001	>0.999[a]	2	1	4.17 (0.11-21.12)		>0.999[a]	4	1	4.17 (0.11-21.12)	<0.001	>0.999[a]
		Medium Dose	24	1	1	4.17 (0.11-21.12)	<0.001	>0.999[b]	0	0	0.00 (0.00-14.25)			1	1	4.17 (0.11-21.12)	<0.001	>0.999[b]
		High Dose	24	0	0	0.00 (0.00-14.25)		>0.999[c]	0	0	0.00 (0.00-14.25)			0	0	0.00 (0.00-14.25)		>0.999[c]
	Constipation	Placebo	24	0	0	0.00 (0.00-14.25)			0	0	0.00 (0.00-14.25)			0	0	0.00 (0.00-14.25)		
		Low Dose	24	1	1	4.17 (0.11-21.12)		>0.999[a]	0	0	0.00 (0.00-14.25)			1	1	4.17 (0.11-21.12)		>0.999[a]
		Medium Dose	24	0	0	0.00 (0.00-14.25)			0	0	0.00 (0.00-14.25)			0	0	0.00 (0.00-14.25)		
		High Dose	24	0	0	0.00 (0.00-14.25)			0	0	0.00 (0.00-14.25)			0	0	0.00 (0.00-14.25)		
	Diarrhoea	Placebo	24	1	1	4.17 (0.11-21.12)			0	0	0.00 (0.00-14.25)			1	1	4.17 (0.11-21.12)		
		Low Dose	24	1	1	4.17 (0.11-21.12)	<0.001	>0.999[a]	2	1	4.17 (0.11-21.12)		>0.999[a]	3	1	4.17 (0.11-21.12)	<0.001	>0.999[a]
		Medium Dose	24	1	1	4.17 (0.11-21.12)	<0.001	>0.999[b]	0	0	0.00 (0.00-14.25)			1	1	4.17 (0.11-21.12)	<0.001	>0.999[b]
		High Dose	24	0	0	0.00 (0.00-14.25)		>0.999[c]	0	0	0.00 (0.00-14.25)			0	0	0.00 (0.00-14.25)		>0.999[c]
Blood and lymphatic system disorders	Summary	Placebo	24	0	0	0.00 (0.00-14.25)			0	0	0.00 (0.00-14.25)			0	0	0.00 (0.00-14.25)		

SOC	PT	Groups	N	Correlation					Irrelevance					Total				
				Number of occurrences	Number of patients	Rate (95% CI)	X^2	*P*	Number of occurrences	Number of patients	Rate (95% CI)	X^2	*P*	Number of occurrences	Number of patients	Rate (95% CI)	X^2	*P*
		Low Dose	24	0	0	0.00 (0.00-14.25)			0	0	0.00 (0.00-14.25)			0	0	0.00 (0.00-14.25)		
		Medium Dose	24	0	0	0.00 (0.00-14.25)			2	2	8.33 (1.03-27.00)	0.522	0.470[b]	2	2	8.33 (1.03-27.00)	0.522	0.470[b]
		High Dose	24	0	0	0.00 (0.00-14.25)			0	0	0.00 (0.00-14.25)			0	0	0.00 (0.00-14.25)		
	Lymphadenitis	Placebo	24	0	0	0.00 (0.00-14.25)			0	0	0.00 (0.00-14.25)			0	0	0.00 (0.00-14.25)		
		Low Dose	24	0	0	0.00 (0.00-14.25)			0	0	0.00 (0.00-14.25)			0	0	0.00 (0.00-14.25)		
		Medium Dose	24	0	0	0.00 (0.00-14.25)			2	2	8.33 (1.03-27.00)	0.522	0.470[b]	2	2	8.33 (1.03-27.00)	0.522	0.470[b]
		High Dose	24	0	0	0.00 (0.00-14.25)			0	0	0.00 (0.00-14.25)			0	0	0.00 (0.00-14.25)		

CI: Confidence Interval.

[a] indicates the low dose group compared with the placebo group.

[b] indicates the medium dose group compared with the placebo group.

[c] indicates the high dose group compared with the placebo group.

Table S4. Analysis of abnormalities in laboratory indicators after full vaccination with vaccine and placebo

Typology	Assay	Groups	N	Number of occurrences	Number of patients	Rate (95% CI)	X^2	*P*
Haematological analyses NEC	Summary	Placebo	24	3	3	12.50 (2.66-32.36)		
		Low Dose	24	3	3	12.50 (2.66-32.36)	<0.001	>0.999[a]
		Medium Dose	24	0	0	0.00 (0.00-14.25)	1.422	0.233[b]
		High Dose	24	2	2	8.33 (1.03-27.00)	<0.001	>0.999[c]
	White blood cell count	Placebo	24	3	3	12.50 (2.66~32.36)		
		Low Dose	24	3	3	12.50 (2.66~32.36)	<0.001	>0.999[a]
		Medium Dose	24	0	0	0.00 (0.00~14.25)	1.422	0.233[b]
		High Dose	24	1	1	4.17 (0.11~21.12)	0.273	0.602[c]
	Lymphocyte count	Placebo	24	0	0	0.00 (0.00~14.25)		
		Low Dose	24	0	0	0.00 (0.00~14.25)		
		Medium Dose	24	0	0	0.00 (0.00~14.25)		
		High Dose	24	1	1	4.17 (0.11~21.12)		>0.999[c]
	Eosinophil count	Placebo	24	0	0	0.00 (0.00~14.25)		
		Low Dose	24	0	0	0.00 (0.00~14.25)		
		Medium Dose	24	0	0	0.00 (0.00~14.25)		
		High Dose	24	0	0	0.00 (0.00~14.25)		
	Platelet count	Placebo	24	0	0	0.00 (0.00~14.25)		
		Low Dose	24	0	0	0.00 (0.00~14.25)		
		Medium Dose	24	0	0	0.00 (0.00~14.25)		
		High Dose	24	0	0	0.00 (0.00~14.25)		
Blood biochemistry	Summary	Placebo	24	3	1	4.17 (0.11~21.12)		
		Low Dose	24	0	0	0.00 (0.00~14.25)		>0.999[a]

Typology	Assay	Groups	N	Number of occurrences	Number of patients	Rate (95% CI)	X^2	*P*
		Medium Dose	24	0	0	0.00 (0.00~14.25)		>0.999[b]
		High Dose	24	1	1	4.17 (0.11~21.12)	<0.001	>0.999[c]
	Alanine aminotransferase	Placebo	24	2	1	4.17 (0.11~21.12)		
		Low Dose	24	0	0	0.00 (0.00~14.25)		>0.999[a]
		Medium Dose	24	0	0	0.00 (0.00~14.25)		>0.999[b]
		High Dose	24	1	1	4.17 (0.11~21.12)	<0.001	>0.999[c]
	Aspartate aminotransferase	Placebo	24	1	1	4.17 (0.11~21.12)		
		Low Dose	24	0	0	0.00 (0.00~14.25)		>0.999[a]
		Medium Dose	24	0	0	0.00 (0.00~14.25)		>0.999[b]
		High Dose	24	0	0	0.00 (0.00~14.25)		>0.999[c]
	Blood bilirubin	Placebo	24	0	0	0.00 (0.00~14.25)		
		Low Dose	24	0	0	0.00 (0.00~14.25)		
		Medium Dose	24	0	0	0.00 (0.00~14.25)		
		High Dose	24	0	0	0.00 (0.00~14.25)		
Urinalysis NEC	Summary	Placebo	24	2	1	4.17 (0.11~21.12)		
		Low Dose	24	1	1	4.17 (0.11~21.12)	<0.001	>0.999[a]
		Medium Dose	24	0	0	0.00 (0.00~14.25)		>0.999[b]
		High Dose	24	3	3	12.50 (2.66~32.36)	0.273	0.602[c]
	Red blood cell	Placebo	24	0	0	0.00 (0.00~14.25)		
		Low Dose	24	1	1	4.17 (0.11~21.12)		>0.999[a]
		Medium Dose	24	0	0	0.00 (0.00~14.25)		
		High Dose	24	1	1	4.17 (0.11~21.12)		>0.999[c]
	Protein urine	Placebo	24	1	1	4.17 (0.11~21.12)		
		Low Dose	24	0	0	0.00 (0.00~14.25)		>0.999[a]
		Medium Dose	24	0	0	0.00 (0.00~14.25)		>0.999[b]
		High Dose	24	2	2	8.33 (1.03~27.00)	<0.001	>0.999[c]
	Glucose urine	Placebo	24	1	1	4.17 (0.11~21.12)		
		Low Dose	24	0	0	0.00 (0.00~14.25)		>0.999[a]

Typology	Assay	Groups	N	Number of occurrences	Number of patients	Rate (95% CI)	X^2	*P*
		Medium Dose	24	0	0	0.00 (0.00~14.25)		>0.999[b]
		High Dose	24	0	0	0.00 (0.00~14.25)		>0.999[c]
Summary		Placebo	24	8	4	16.67 (4.74~37.38)		
		Low Dose	24	4	4	16.67 (4.74~37.38)	<0.001	>0.999[a]
		Medium Dose	24	0	0	0.00 (0.00~14.25)	2.455	0.117[b]
		High Dose	24	6	5	20.83 (7.13~42.15)	<0.001	>0.999[c]

CI: Confidence Interval.

[a] indicates the low dose group compared with the placebo group.

[b] indicates the medium dose group compared with the placebo group.

[c] indicates the high dose group compared with the placebo group.

Table S5. Adverse reactions and incidence sorted by type 0-7 days after full vaccination in the 6-17 age group

	**Placebo (N=24)**		**Low Dose (N=24)**		**Medium Dose (N=24)**		**High Dose (N=24)**	
	**N (%)**	**No. of Event**	**N (%)**	**No. of Event**	**N (%)**	**No. of Event**	**N (%)**	**No. of Event**
**Total adverse reactions**	2 (8.33)	3	9 (37.50)	12	3 (12.50)	5	1 (4.17)	2
**Localized adverse reaction**	1 (4.17)	1	6 (25.00)	6	1 (4.17)	2	1 (4.17)	2
**Systemic adverse reaction**	2 (8.33)	2	4 (16.67)	6	3 (12.50)	3	0	0
**Level 3 and above adverse reactions**	0	0	0	0	0	0	0	0

Table S6. Comparison of rotavirus neutralizing antibody GMT on day 28 after full immunization in the adolescent group

Groups	N-valid	N-missing	Pre-immunization GMT (95% CI)	GMT (95% CI)	GMI (95% CI)	Seroconversion rate (% with 95% CI)
Placebo	24	0	197.20 (103.70 to 375.01)	270.95 (155.53 to 472.00)	1.37 (1.11 to 1.70)	0.00 (0.00 to 14.25)
Low dose	24	0	228.05 (119.92 to 433.69)	424.32 (283.05 to 636.10)	1.86 (1.24 to 2.79)	8.33 (1.03 to 27.00)
Medium dose	24	0	234.84 (123.49 to 446.59)	504.63 (358.79 to 709.76)	2.15 (1.45 to 3.18)	33.33 (15.63 to 55.32)
High dose	24	0	259.45 (136.43 to 493.40)	925.45 (708.10 to 1209.52)	3.57 (2.84 to 4.48)	54.17 (32.82 to 74.45)

Note: Neutralizing antibody positive transfer refers to negative pre-immunization antibody and positive post-immunization antibody (an antibody potency less to 1:8 is considered negative, while that greater than or equal to 1:8 is considered positive), or else positive pre-immunization antibody and a 4-fold increase in antibody level after immunization compared to the baseline value.

Table S7. Comparison of GMT of serum rotavirus-specific IgG on day 28 after full immunization in the adolescent group

Group	N-valid	N-missing	Pre-immunization GMT (95% CI)	GMT (95% CI)	GMI (95% CI)	Seroconversion rate (% with 95% CI)
Placebo	24	0	745.06 (403.75 to 1374.92)	837.09 (474.19 to 1477.71)	1.12 (0.87 to 1.46)	4.17 (0.11 to 21.12)
Low dose	24	0	1009.25 (546.91 to 1862.44)	6501.86 (4047.23 to 10445.2)	6.44 (3.64 to 11.40)	62.50 (40.59 to 81.20)
Medium dose	24	0	952.82 (516.33 to 1758.31)	6501.82 (3718.35 to 11368.9)	6.82 (4.40 to 10.57)	79.17 (57.85 to 92.87)

Group	N-valid	N-missing	Pre-immunization GMT (95% CI)	GMT (95% CI)	GMI (95% CI)	Seroconversion rate (% with 95% CI)
High dose	24	0	848.47 (459.78 to 1565.75)	10173.3 (4512.65 to 22934.8)	11.99 (5.73 to 25.09)	91.67 (73.00 to 98.97)

Note: Positive IgG antibody conversion refers to negative pre-immunization antibody and positive post-immunization antibody (an antibody potency less than or equal to 1:16 is considered negative, while that greater than to 1:16 is considered positive), or else positive pre-immunization antibody and a 4-fold increase in antibody level after immunization compared to the baseline value.

Table S8. Comparison of GMT of serum rotavirus-specific IgA on day 28 after full immunization in the adolescent group

Group	N-valid	N-missing	Pre-immunization GMT (95% CI)	GMT (95% CI)	GMI (95% CI)	Seroconversion rate (% with 95% CI)
Placebo	24	0	443.12 (244.13 to 804.31)	400.79 (255.90 to 627.72)	0.90 (0.75 to 1.09)	0.00 (0.00 to 14.25)
Low dose	24	0	654.56 (360.62 to 1188.09)	2733.64 (1465.57 to 5098.91)	4.18 (2.37 to 7.37)	54.17 (32.82 to 74.45)
Medium dose	24	0	418.25 (230.43 to 759.16)	2233.29 (1231.49 to 4050.03)	5.34 (2.71 to 10.53)	70.83 (48.91 to 87.38)
High dose	24	0	362.18 (199.54 to 657.39)	3596.66 (1957.20 to 6609.41)	9.93 (5.49 to 17.97)	79.17 (57.85 to 92.87)

Note: Positive IgA antibody conversion refers to negative pre-immunization antibody and positive post-immunization antibody (an antibody potency less to 1:8 is considered negative, while that greater than or equal to 1:8 is considered positive), or else positive pre-immunization antibody and a 4-fold increase in antibody level after immunization compared to baseline the value.
